# Block periodization vs. traditional periodization in high-intensity functional training: a randomized crossover study

**DOI:** 10.3389/fphys.2026.1810477

**Published:** 2026-05-20

**Authors:** Florian Micke, Julius Huelle, Gommaar D’Hulst, Eduard Isenmann, Stephan Geisler, Steffen Held

**Affiliations:** 1 Department of Sport and Management, IST University of Applied Sciences, Duesseldorf, Germany; 2 Department of Health Sciences and Technology, ETH Zurich, Zurich, Switzerland; 3 Department of Fitness and Health, IST University of Applied Sciences, Duesseldorf, Germany

**Keywords:** training organization, crossover, functional fitness, maximal strength, metabolic conditioning

## Abstract

**Background:**

High-intensity functional training (HIFT) requires the combined development of strength and metabolic conditioning. This study examined whether concentrating high-intensity training loads into specific blocks provides advantages over a traditional evenly distributed approach in experienced athletes.

**Methods:**

Twenty experienced HIFT athletes (34.5 ± 9.8 yrs, 80.9 ± 13.7 kg, 1.77 ± 0.11 m, training experience: 5.8 ± 3.2 yrs) completed two six-week, load-matched intervention phases (block periodization (BP) versus traditional periodization (TP)) in a randomized controlled crossover design. Both conditions included five weekly sessions, but differed in the temporal distribution of high-intensity training (HIT) and low-intensity training (LIT) sessions, with BP concentrating HIT sessions into specific loading weeks and TP distributing intensity more evenly across the intervention. Outcome measures included maximal strength, benchmark workouts, physiological stress assessed via countermovement jump (CMJ), and health-related parameters including resting blood pressure, blood glucose concentration, and body composition.

**Results:**

BP was associated with greater improvements in maximal strength compared to TP. Specifically, significant group differences favoring BP were observed for the isometric mid-thigh pull (p = 0.038, d = 0.82), deadlift one repetition maximum (p = 0.018, d = 0.85), and CrossFit^®^ Total (p = 0.027, d = 0.78). Furthermore, BP resulted in a significant reduction in waist circumference (p = 0.019, d = -0.79), suggesting a favorable anthropometric change. Monitoring data revealed a significant acute reduction in CMJ during the concentrated loading week of BP (p < 0.001, d = -1.41), consistent with a state of functional overreaching. No significant between-condition differences were observed for metabolic conditioning, resting blood pressure and blood glucose concentration.

**Conclusion:**

Concentrated loading through block periodization was associated with favorable changes in selected maximal strength outcomes in trained HIFT athletes without compromising metabolic conditioning or cardiovascular health markers. This approach effectively induces functional overreaching and represents a viable strategy to optimize performance adaptations in HIFT.

## 1 Introduction

CrossFit^®^, frequently characterized as high-intensity functional training (HIFT), has rapidly developed into a globally significant fitness trend and sport (Claudino et al., 2018; Dominski et al., 2022; Feito et al., 2018; Schlegel, 2020). This multimodal training methodology intentionally employs constantly varied functional movements performed at high-intensity, systematically integrating elements from weightlifting, gymnastics, and intense metabolic conditioning (Claudino et al., 2018; Martinho et al., 2024; Schlegel, 2020). The primary objective is to develop broad and inclusive fitness across multiple domains, including cardiovascular/respiratory endurance, stamina, strength, flexibility, power, speed, coordination, agility, balance and accuracy (Glassman, 2002). Subsequent studies have empirically examined this multidimensional fitness concept within HIFT populations (Ambroży et al., 2022; Claudino et al., 2018; Held et al., 2024; Rios et al., 2024).

The highly demanding, mixed-modal nature of functional fitness requires substantial physiological responses (Martinho et al., 2024). Specifically, competitive CrossFit^®^ workouts (WODs) frequently elicit extreme metabolic demands, characterized by maximum or near-maximum heart rates (HR) and significantly elevated blood lactate concentrations (Forte et al., 2022; Jacob et al., 2020; Leitão et al., 2021; Martinho et al., 2024). While this intensive training approach efficiently improves physical performance, it simultaneously imposes a high cumulative training load (Schlegel, 2020), which can lead to transient muscle damage (Leite et al., 2023), increased creatine kinase levels, neuromuscular fatigue (Martinho et al., 2024), and, in some cases, severe complications such as exertional rhabdomyolysis (Adhikari et al., 2021; Lawrensia et al., 2021). Therefore, optimizing the organization of training and ensuring sufficient recovery is paramount for competitive success and sustained athlete health (Jacob et al., 2020). In addition, acute responses to functional fitness sessions appear to depend strongly on session intensity and structure. Tibana et al. (2022) demonstrated that functional fitness sessions performed at different intensities induce distinct time-course responses in metabolic, hormonal, and neurotrophic markers, further emphasizing the importance of intensity management and recovery planning in HIFT. From an applied perspective, Pritchard et al. (2020) reported that elite CrossFit^®^ athletes used tapering strategies before important competitions, typically reducing training volume and manipulating strength and conditioning loads during the weeks preceding competition. These findings indicate that intensity distribution and load management are highly relevant in HIFT practice, but experimental evidence comparing distinct periodization models in experienced HIFT athletes remains limited. In endurance sports, researchers frequently contrast different strategies for long-term load management, particularly focusing on Block Periodization (BP) and Traditional Periodization (TP) (Mølmen et al., 2019; Wetmore et al., 2020). TP models typically distribute high-intensity training (HIT), moderate-intensity training (MIT), and low-intensity training (LIT) relatively evenly across a weekly microcycle (Mølmen et al., 2019; Solli et al., 2019). In contrast, BP structures training into concentrated blocks (Issurin, 2008), deliberately prioritizing the successive development of specific abilities by temporally clustering multiple HIT sessions within a given training week, followed by weeks with reduced HIT exposure, rather than distributing intensity evenly across all weeks (Mølmen et al., 2019; Wetmore et al., 2020).

Studies involving highly trained endurance athletes, such as cyclists and skiers, indicate that concentrated BP microcycles can be an effective strategy, often yielding a small but superior enhancement in maximal aerobic capacity (V̇O2max) and power output compared to traditional methods (Breil et al., 2010; Mølmen et al., 2019; Rønnestad et al., 2022). However, the literature is not entirely consistent, with some research finding that superior physiological benefits observed in BP do not always translate to differential performance outcomes when overall load is strictly matched, or observing no additional benefits compared to evenly distributed HIT (Almquist et al., 2022; McGawley et al., 2017).

Despite the strong conceptual overlap between functional fitness and concurrent strength and endurance training principles (Dominski et al., 2022; Schlegel, 2020), limited research has directly applied and systematically compared structured periodization models, such as BP, within the highly specific, mixed-modal context of experienced HIFT athletes (Martinho et al., 2024). We need evidence-based training recommendations that integrate the complex strength, power, and metabolic demands of this sport, considering that training volume and intensity are critical drivers of performance gains (Mangine et al., 2020). Furthermore, it remains critically unclear whether concentrating high-intensity metabolic conditioning loads in experienced HIFT athletes, while maintaining total training volume, is superior to a more evenly distributed approach.

Previous HIFT interventions have examined general training-induced adaptations and acute responses to workouts of different intensities. However, direct comparisons of block-periodized and evenly distributed high-intensity training structures remain limited in experienced HIFT athletes. Thus, this randomized, load-matched crossover study addresses this methodological gap by comparing the effects of six weeks of BP against TP in experienced HIFT athletes. This experimental design specifically manipulated the temporal distribution of HIT, focusing either on concentrated high-load blocks (BP) or an evenly distributed intensity structure (TP). We hypothesized that BP would lead to greater improvements in selected performance outcomes than TP due to the concentrated organization of high-intensity training stimuli, while maintaining metabolic conditioning capacity.

## 2 Materials and methods

### 2.1 Participants

Based on an *a priori* sample size calculation, we determined the required number of participants using G*Power software (Version 3.1.9.7) (Kang, 2021). We selected an F-test for a repeated measures ANOVA with within-subject factors to reflect the study’s design. To detect a medium effect size (f =0.25) given a significance level (α) of 0.05 and a statistical power (1−β) of 0.80, while assuming a correlation among repeated measures of 0.75 for repeated measurement points, the analysis indicated that a total sample size of 18 participants was required. Participants were recruited from a local CrossFit^®^/HIFT training facility via on-site announcements, email newsletter and personal contact. Interested athletes received written and verbal information about the study procedures and were screened for eligibility according to the predefined inclusion and exclusion criteria. Assuming moderate dropouts, 20 HIFT athletes (male: n = 9, 37.3 ± 10.2 yrs, 90.8 ± 12.3 kg, 1.86 ± 0.08 m, HIFT experience: 4.9 ± 2.9 yrs) (female: n = 11, 32.3 ± 9.4 yrs, 71.0 ± 5.5 kg, 1.69 ± 0.06 m, HIFT experience: 6.6 ± 3.5 yrs) participated in this study. Male participants demonstrated mean relative back squat and deadlift strength values of 1.43 ± 0.25 and 1.84 ± 0.22 1RM/BM, respectively, while female participants demonstrated corresponding values of 1.56 ± 0.23 and 1.85 ± 0.34 1RM/BM. Based on the strength-level criteria proposed by Santos Junior et al. (2021), these values correspond predominantly to advanced or highly advanced lower-body strength levels.

We defined inclusion criteria as a minimum age of 18 years, at least one year of extreme conditioning program training and competition experience, a minimum of five weekly training sessions over the three months preceding the study and the absence of musculoskeletal injuries in the four weeks prior to the study. We excluded individuals with acute or chronic orthopedic, internal, or neurological conditions that would contraindicate high-intensity physical exertion. All participants provided written informed consent. The study protocol complied with the Declaration of Helsinki and was approved by the Local Ethical Committee (144/2022) and fulfilled the international ethical standards (Harriss and Atkinson, 2015).

### 2.2 Research design

**Figure 1:**
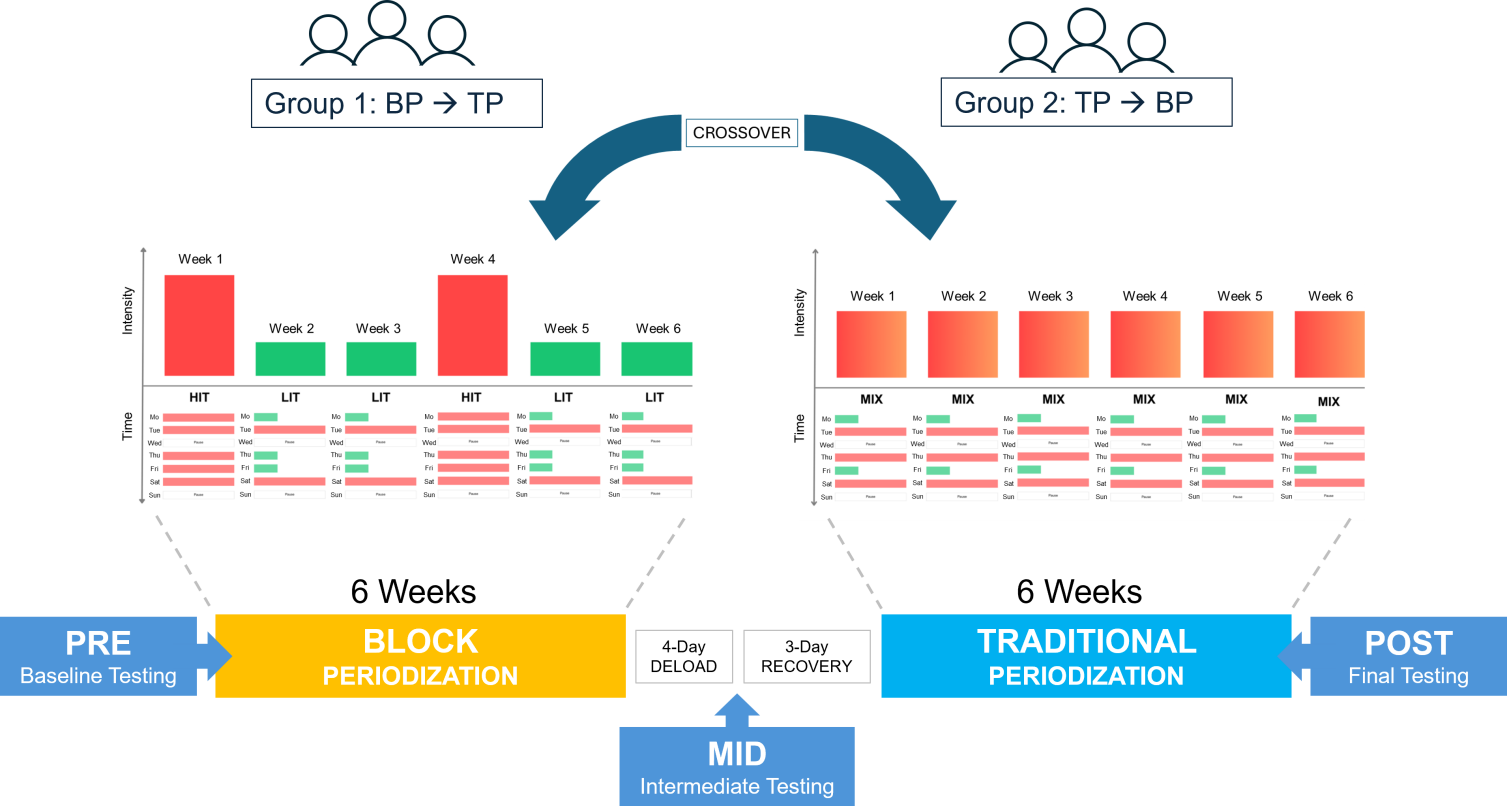
Schematic representation of the study protocol. BP, block periodization; TP, traditional periodization; HIT, high-intensity training; LIT, low-intensity training.

A randomized controlled crossover design was employed to compare the effects of BP versus TP on physical performance and health parameters. Participants were randomly assigned to one of two sequences, starting with either the BP protocol or the TP protocol. The crossover design included two six-week intervention phases (**Figure 1**). The washout period between the two intervention phases consisted of a four-day deload phase, followed by the intermediate diagnostics and a subsequent three-day recovery phase to minimize fatigue effects before the second intervention block commenced. Data were collected at three specific time points: before the first intervention (PRE), after the first intervention (MID), and after completion of the second intervention (POST). All measurements were conducted at comparable times of day for each participant to account for potential circadian influences on physiological measures and performance. Environmental conditions, including temperature and humidity, were kept constant throughout all testing sessions. Participants were instructed to refrain from intense training for at least 72 h prior to each test.

### 2.3 Intervention

The training intervention focused specifically on metabolic conditioning workouts and supplementary endurance sessions. Strength training was not part of the prescribed intervention. However, participants received an identical strength training plan prior to the study and were instructed to continue this program independently, either in supervised classes or on their own, while no additional monitoring procedures were implemented for these sessions.

Regardless of group assignment, all participants completed a fixed weekly structure of five sessions per week, consisting of three metabolic conditioning (MetCon) workouts and two running sessions. By the end of the intervention, the BP and TP groups performed the exact same pool of workouts with an identical total training volume. However, due to the different periodization models, the temporal organization of the HIT and LIT workouts differed. Over the six-week period, the TP group distributed the workouts evenly, performing three HIT and two LIT workouts per week. In contrast, the BP group utilized two three-week cycles. Each cycle commenced with a concentrated week of five HIT sessions followed by two weeks containing only two HIT and three LIT sessions. A detailed overview of the complete study schedule and training intervention, including the weekly HIT/LIT distribution, all workout descriptions and the strength plan, is provided in Supplementary File 1.

High-intensity training (HIT) sessions were defined as workouts performed at a rating of perceived exertion (RPE) of eight or higher on the modified Borg scale. HIT protocols employed various formats, including interval-based sessions, “For Time” workouts, and “Chippers”. A representative HIT MetCon consisted of four 8-minute blocks (6 minutes of work, 2 minutes of rest), combining 400m runs with high-demand movements such as sandbag-to-shoulders, double unders, and a maximal-effort of complex gymnastic exercises (e.g., muscle-ups or handstand push-ups). Similarly, HIT running sessions involved track intervals (e.g., sets of 800m, 600m, and 400m) performed at intensities exceeding the participant’s 5k race pace.

Low intensity training (LIT) sessions targeted an RPE below five. A representative LIT MetCon was performed as a 30-minute EMOM (Every Minute on the Minute), incorporating exercises such as bar-facing burpees, hang snatches, rowing, and rope climbs, with one minute of rest between rounds. LIT running sessions focused on aerobic base development, with intensity maintained within low RPE zones (RPE 2-4) and, when necessary, interspersed with walking breaks.

### 2.4 Diagnostics

During the diagnostic weeks, a comprehensive battery of tests was conducted to assess health markers, body composition, and sport-specific performance. On Day 1, participants completed all health related diagnostics (blood pressure, blood glucose, body composition), the Isometric mid-thigh pull (IMTP) and the Countermovement jump (CMJ); on Day 2 the endurance workout (AMRAP Burpees & rowing; on Day 3 the CrossFit^®^ Total; on Day 4 the 1RM clean and jerk; on Day 5 the mixed-modal workout; on Day 6 the power workout; and on Day 7 the step test. This standardized sequence was maintained across PRE, MID, and POST.

All health-related assessments were performed during a single standardized appointment. Resting systolic and diastolic blood pressure were measured using an automated monitor (BIG 5611, AEG, Germany), and blood glucose concentration was determined via a single capillary blood sample (Contour Next, Ascensia Diabetes Care Germany GmbH, Germany). Body composition, including body weight, fat percentage, and skeletal muscle mass, was assessed using bioelectrical impedance analysis (seca mBCA Go, seca Germany gmbh, Germany). Participants were asked to arrive in a rested and normally hydrated condition, refrain from large meals and excessive fluid intake immediately before bioelectrical impedance analysis and empty their bladder shortly before the assessment. Waist circumference was measured manually using a non-elastic measurement tape (seca 201, seca Germany gmbh, Germany).

Physical performance was evaluated using a series of CrossFit^®^-specific tests conducted over six consecutive days and followed the same standardized order at each diagnostic time point to minimize variability caused by test order. All measurements were made in the same testing environment at comparable times of the day. All tests were supervised by certificated coaches. No standardized warm-up protocol was prescribed. Participants performed an individualized self-selected warm-up before each performance test, reflecting common HIFT practice and allowing experienced athletes to prepare according to their habitual routines.

Isometric mid-thigh pull (IMTP) peak force (Grgic et al., 2022) was assessed using a Tindeq force sensor (Tindeq ProGessor, BLIMS AS, Norway). Countermovement jump (CMJ) height (mean of best 2 jumps of 3) was measured using the validated MyJump2 application (MyJump2, Carlos Balsalobre-Fernández, Spain) (Pueo et al., 2023; Stanton et al., 2017; Vieira et al., 2023). Maximal strength was determined through the CrossFit^®^ Total. The CrossFit^®^ Total was assessed as the sum of the highest successfully completed 1RM attempts in the back squat, strict press and deadlift. In accordance with the traditional format, athletes performed the lifts in the standardized order of back squat, strict press and deadlift. For each exercise, participants were allowed up to three maximal attempts within a 20-minute time window. A lift was considered valid only when the predefined technical standards were achieved. The highest valid load for each lift was recorded and the sum of the three lifts was used as the CrossFit^®^ Total score. In addition, the 1RM for the clean and jerk was assessed separately. Participants completed the test within a 20-minute time window, and the highest technically valid lift was recorded.

Metabolic conditioning and work capacity were evaluated using four distinct workouts. The first test combined a seven-minute AMRAP (as many reps/rounds as possible) of burpees, immediately followed by a 1000m rowing time trial to assess HIFT-specific mixed-modal work capacity, in which high-repetition bodyweight exercise is followed by a cyclical endurance task. Performance outcomes were the total number of burpees completed (Burpees WOD) and the rowing time. The second test was a power-oriented workout (Power WOD) consisting of a 21-15–9 repetition scheme of deadlifts and calories on an Assault Bike, with total time to completion recorded. The third assessment comprised a mixed-modal workout requiring participants to complete a fixed workload of rowing, toes to bar, wall ball shots, cleans, and ring muscle ups within a 14-minute time cap. Outcomes included the total number of repetitions completed (Mixed Modal WOD) and the tiebreak time recorded after the cleans (Tiebreak WOD). Finally, a step test (Step Test WOD) involving increasing intervals of thrusters and burpees was performed to determine threshold performance based on total repetitions achieved.

### 2.5 Monitoring

All training prescriptions and diagnostic protocols were delivered via a digital training application (Fuse Method, NordAthletik GmbH, Germany). The platform was used to communicate detailed workout instructions and prescribed intensity targets. To monitor weekly fluctuations in neuromuscular fatigue and recovery status, participants additionally performed a CMJ test (Claudino et al., 2017) and completed the Hooper Index questionnaire (Hooper et al., 1995). Training adherence was monitored by tracking completion of assigned sessions and evaluating compliance with the prescribed high- and low-intensity distributions. A minimum adherence of 80% of scheduled training sessions was required for inclusion in the final analysis.

### 2.6 Statistics

All statistical analyses were performed using the R computing environment (RStudio version 2023.06.0) with the lme4, lmerTest, and tidyverse packages. The normality of data distribution was assessed using the Shapiro-Wilk test and the assumption of sphericity was checked using Mauchly’s test. Upon visual inspection of residual plots and statistical testing, we identified several variables that violated this normality assumption. Consequently, we applied a logarithmic transformation to Blood Glucose, Skeletal Muscle Mass, Strict Press strength, Rowing performance, Power Workout duration, Step Test repetitions, and the Tiebreak time to approximate a normal distribution before further analysis. To evaluate the comparative effectiveness of the interventions, we employed Linear Mixed Models (LMM). This statistical approach specifically accounts for the repeated measures structure inherent in the randomized crossover design. We modeled the change scores (delta) from pre-intervention to post-intervention as the dependent variable. The model specification included the intervention type (BP vs. TP), the study period, and the allocation sequence as fixed effects to control for potential order or carryover effects. Furthermore, the baseline value of each respective outcome was included as a fixed covariate to adjust for initial differences and regression to the mean. We treated the individual participant identity as a random effect to account for between-subject variability. For the analysis of weekly monitoring data (CMJ height and Hooper Index), we fitted separate LMM to assess time-course changes. These models included ‘Intervention’ (BP vs. TP), ‘Week’ (treated as a categorical factor), and their interaction term as fixed effects, with participant identity modeled as a random intercept to account for within-subject correlation. To dissect potential differences at specific time points, we performed pairwise *post-hoc* comparisons between groups for each week using estimated marginal means (emmeans package). Pairwise effect sizes (Cohen’s d) for these weekly comparisons were calculated by dividing the estimated mean difference by the model’s residual standard deviation. The magnitude of the differences between the training protocols was quantified using Cohen’s d effect sizes calculated from the unadjusted mean differences in change scores and the pooled standard deviation (Cohen, 2013). We interpreted the magnitude of these effects according to standard thresholds where values less than 0.20 indicated a trivial effect, values between 0.20 and 0.50 represented a small effect, values between 0.50 and 0.80 denoted a medium effect, and values greater than 0.80 signified a large effect (Cohen, 2013). We set the threshold for statistical significance at an alpha level of 0.05 for all comparisons and reported descriptive data as means and standard deviations.

## 3 Results

### 3.1 Performance related outcomes

**Figure 2:**
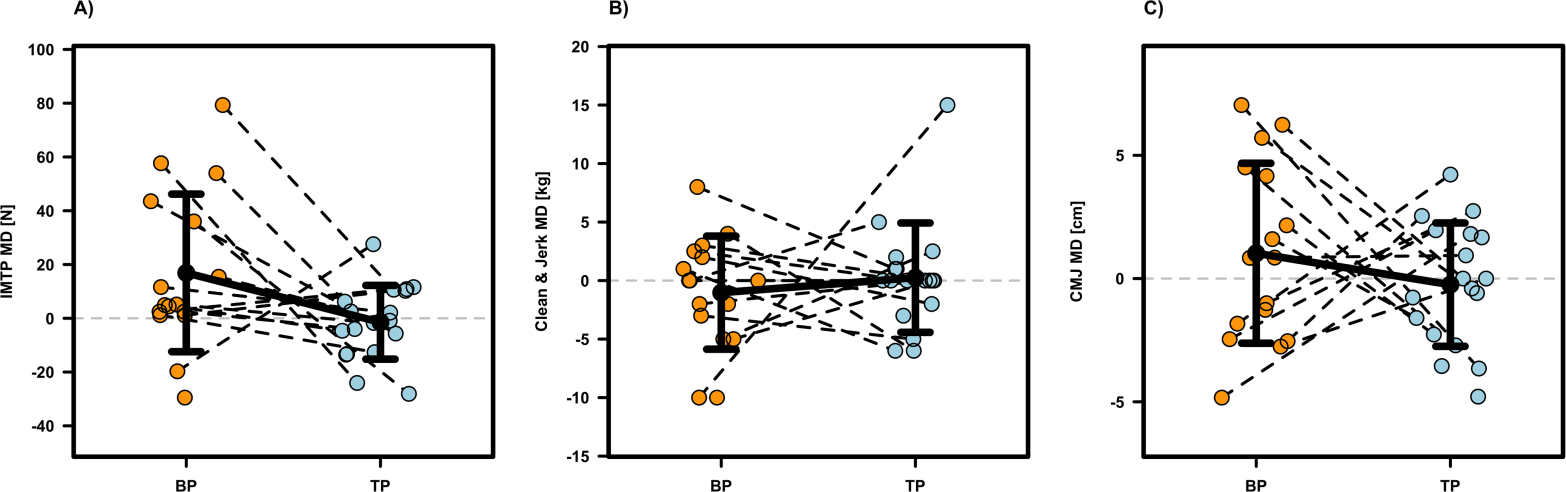
Individual and mean difference (MD) for Isometric Mid-Thigh Pull [**(A)**, IMTP], clean & jerk one repetition maximum **(B)** and countermovement jump height [**(C)**, CMJ]. The plots display individual data points (colored dots) for Block Periodization (BP, orange) and Traditional Periodization (TP, light blue). Black crossbars indicate the group mean difference ± standard deviation.

**Figure 3:**
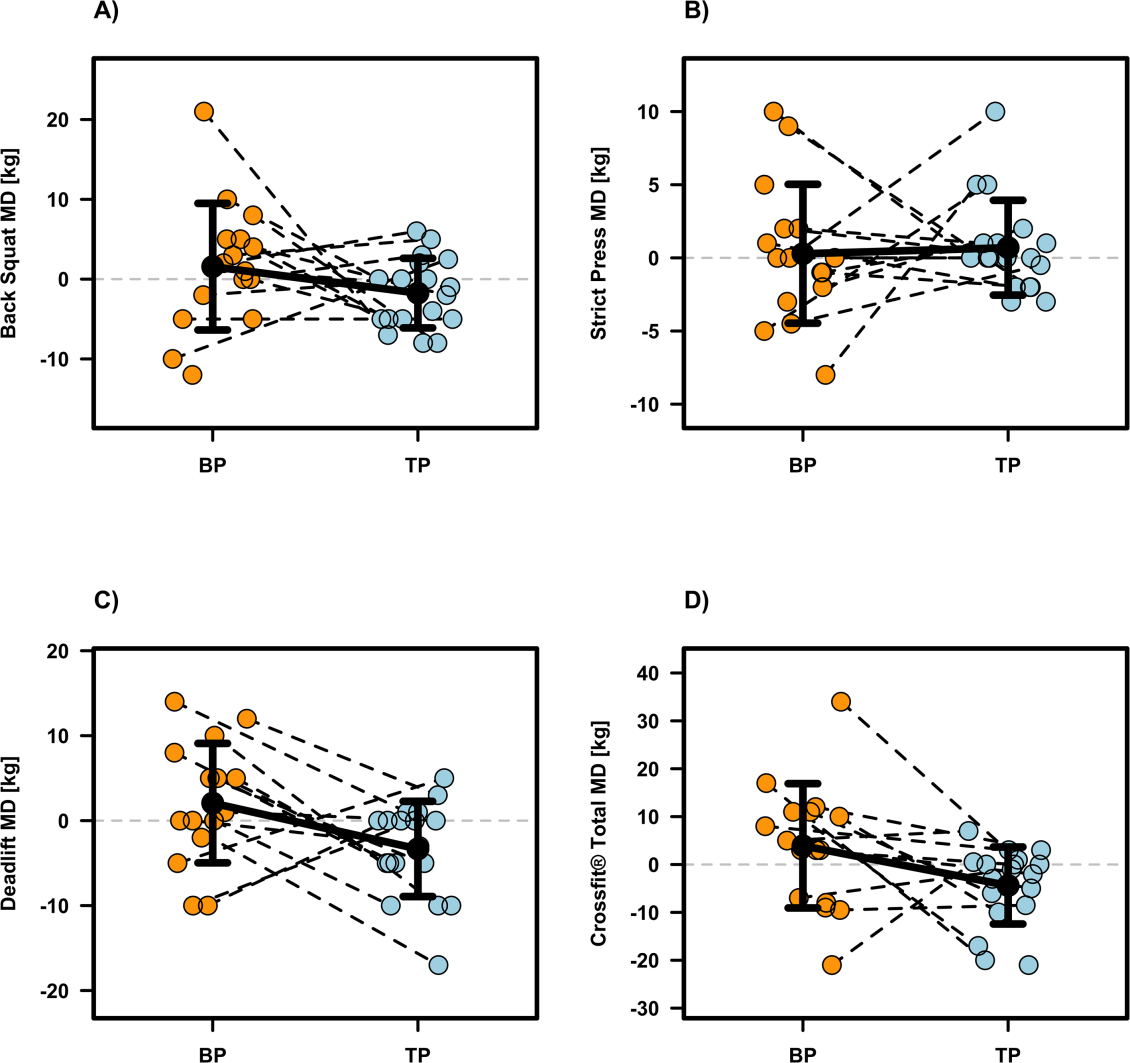
Individual and mean difference (MD) for one repetition maximum (1RM) Back Squat **(A)**, Strict Press 1RM **(B)**, Deadlift 1RM **(C)** and CrossFit^®^ Total **(D)**. The plots display individual data points (colored dots) for Block Periodization (BP, orange) and Traditional Periodization (TP, light blue). Black crossbars indicate the group mean difference ± standard deviation.

**Figure 4:**
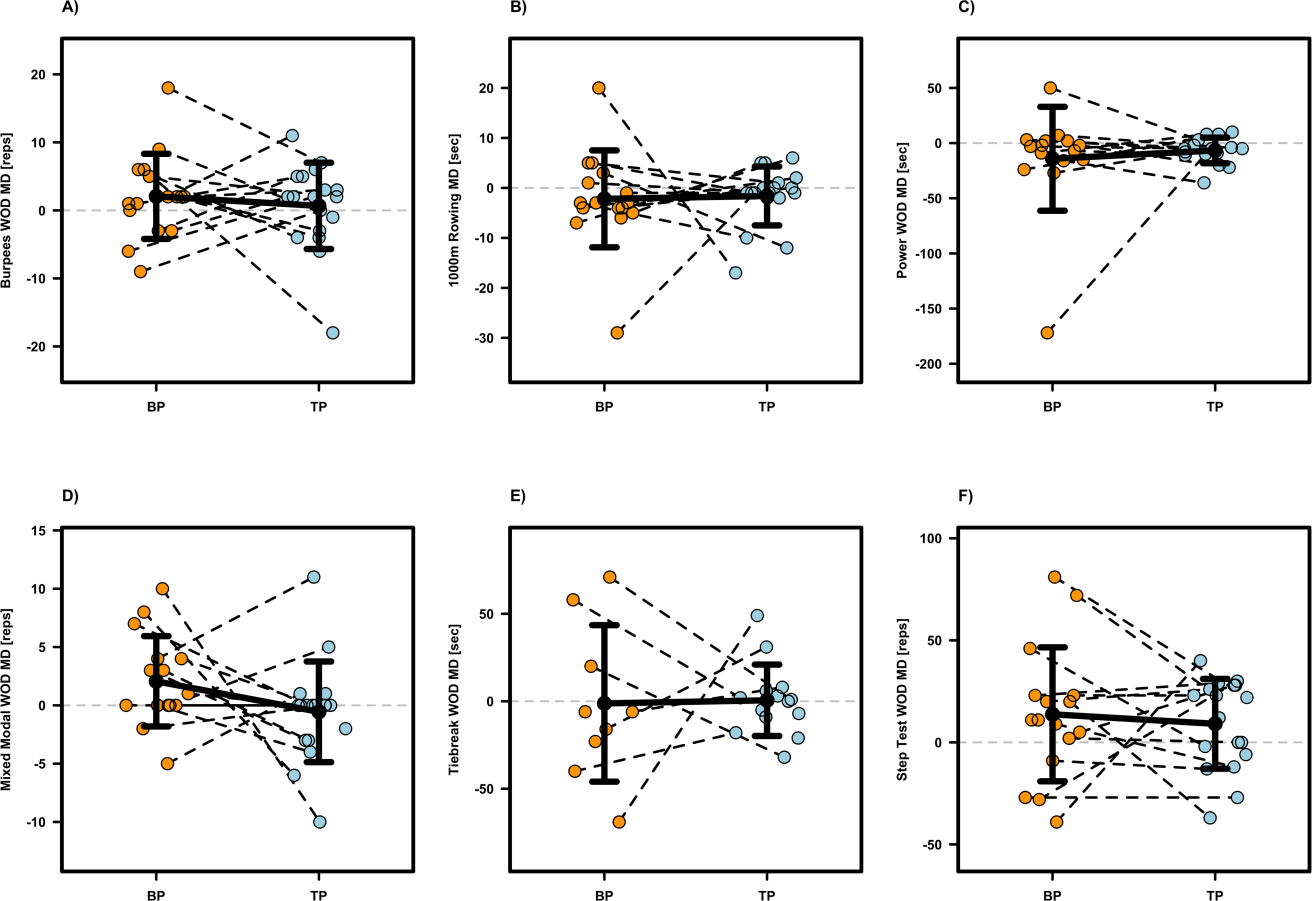
Individual and mean difference (MD) for Burpess WOD **(A)**, 1000m Rowing WOD **(B)**, Power WOD **(C)**, Mixed Modal WOD **(D)**, Tiebreak WOD **(E)** and Step Test WOD **(F)**. The plots display individual data points (colored dots) for Block Periodization (BP, orange) and Traditional Periodization (TP, light blue). Black crossbars indicate the group mean difference ± standard deviation.

Most performance related outcomes revealed no significant intervention effects (p ≥0.104; see **Table 1**), except for IMTP (p =0.038, β =176.08, SE = 80.87, d =0.82), deadlift (p =0.018, β =5.53, SE = 2.11, d =0.85), and CrossFit^®^ Total (p =0.027, β =8.64, SE = 3.71, d =0.78), which were significantly in favor of BP compared to TP. Individual participant data and mean differences are presented in **Figures 2**–**4**. Although not statistically significant, moderate effect sizes favoring the BP protocol were also observed for the Back Squat (p =0.143, β =3.46, SE = 2.29, d =0.53) and the Mixed-Modal WOD (p =0.104, β =2.43, SE = 1.44, d =0.64). Regarding the Step Test WOD, a significant baseline effect was observed (β =−0.146, SE = 0.048, p =0.003), demonstrating a negative relationship between initial performance and subsequent improvement. Thus, athletes with lower baseline scores achieved relatively greater gains. Furthermore, the Step Test WOD showed a significant sequence effect (β =0.080, SE = 0.031, p =0.010), suggesting that the intervention order influenced the magnitude of performance changes, with the group starting with TP showing a greater overall improvement trajectory. Apart from these findings, no significant effects for baseline values or the sequence of interventions were found for any other performance-related outcome (see **Table 1**).

**Table 1:** Performance and health-related outcomes for Block Periodization (BP) versus Traditional Periodization (TP).

	Block Periodization (BP)		Traditional Periodization (TP)		LMM Results		
	Mean ± SD	MD ± SD	Mean ± SD	MD ± SD	Intervention	Baseline	Sequence
Performance related outcomes:							
IMTP [N]	2053.29 ± 544.25	165.57 ± 287.51	2157.26 ± 574.88	-14.56 ± 134.55	**p = 0.038, β = 176.083, SE = 80.866, MD = 180.13 ± 219.92, d = 0.82**	p = 0.952, β = 0.004, SE = 0.073	p = 0.815, β = -18.984, SE = 80.532
Clean & Jerk [kg]	80.09 ± 21.37	-1.03 ± 4.83	87.31 ± 24.54	0.25 ± 4.67	p = 0.396, β = -1.455, SE = 1.690, MD = -1.28 ± 4.75, d = -0.27	p = 0.863, β = 0.007, SE = 0.039	p = 0.731, β = -0.622, SE = 1.790
CMJ [cm]	34.26 ± 8.48	1.03 ± 3.65	36.44 ± 7.48	-0.25 ± 2.50	p = 0.317, β = 1.009, SE = 0.990, MD = 1.27 ± 3.09, d = 0.41	p = 0.413, β = -0.055, SE = 0.067	p = 0.169, β = -1.475, SE = 1.045
Back Squat [kg]	115.00 ± 26.18	1.56 ± 7.93	121.83 ± 26.70	-1.75 ± 4.36	p = 0.143, β = 3.457, SE = 2.294, MD = 3.31 ± 6.29, d = 0.53	p = 0.729, β = -0.016, SE = 0.045	p = 0.500, β = 1.617, SE = 2.366
Press* [kg]	54.34 ± 14.24	0.28 ± 4.75	56.17 ± 15.39	0.69 ± 3.24	p = 0.771, β = -0.008, SE = 0.028, MD = -0.41 ± 4.02, d = -0.10	p = 0.360, β = -0.048, SE = 0.052	p = 0.968, β = 0.001, SE = 0.028
Deadlift [kg]	142.31 ± 37.21	2.06 ± 7.03	150.06 ± 35.61	-3.33 ± 5.60	**p = 0.018, β = 5.525, SE = 2.110, MD = 5.40 ± 6.31, d = 0.85**	p = 0.955, β = 0.002, SE = 0.031	p = 0.456, β = 1.693, SE = 2.219
CrossFit^®^ Total [kg]	311.66 ± 74.67	3.91 ± 12.98	328.06 ± 75.25	-4.39 ± 8.04	**p = 0.027, β = 8.644, SE = 3.705, MD = 8.30 ± 10.65, d = 0.78**	p = 0.865, β = 0.004, SE = 0.025	p = 0.385, β = 3.302, SE = 3.743
Burpees WOD [reps]	107.75 ± 16.82	2.06 ± 6.26	108.94 ± 15.64	0.67 ± 6.34	p = 0.356, β = 2.004, SE = 2.137, MD = 1.40 ± 6.30, d = 0.22	p = 0.115, β = -0.118, SE = 0.073	p = 0.057, β = 4.617, SE = 2.327
1000m Rowing* [s]	235.62 ± 20.56	-2.19 ± 9.70	232.44 ± 21.27	-1.61 ± 5.89	p = 0.991, β = 0.000, SE = 0.011, MD = -0.58 ± 7.91, d = -0.07	p = 0.123, β = -0.100, SE = 0.063	p = 0.411, β = 0.009, SE = 0.011
Power WOD* [s]	337.07 ± 118.01	-14.20 ± 47.10	314.65 ± 98.04	-6.65 ± 11.60	p = 0.729, β = -0.008, SE = 0.023, MD = -7.55 ± 33.27, d = -0.23	p = 0.205, β = -0.069, SE = 0.052	p = 0.892, β = -0.004, SE = 0.028
Mixed Modal WOD [reps]	178.75 ± 16.25	2.06 ± 3.87	184.33 ± 20.20	-0.56 ± 4.31	p = 0.104, β = 2.426, SE = 1.443, MD = 2.62 ± 4.11, d = 0.64	p = 0.114, β = -0.076, SE = 0.046	p = 0.507, β = 1.136, SE = 1.690
Tiebreak WOD* [s]	707.67 ± 74.17	-1.22 ± 44.73	687.29 ± 99.51	0.57 ± 20.41	p = 0.878, β = -0.003, SE = 0.021, MD = -1.79 ± 31.94, d = -0.06	p = 0.992, β = -0.002, SE = 0.176	p = 0.887, β = 0.006, SE = 0.043
Step Test WOD* [reps]	343.56 ± 115.23	13.75 ± 32.82	356.72 ± 109.49	9.11 ± 22.00	p = 0.364, β = 0.025, SE = 0.027, MD = 4.64 ± 27.61, d = 0.17	**p = 0.013, β = -0.146, SE = 0.053**	**p = 0.030, β = 0.080, SE = 0.034**
Health related outcomes:							
Systolic Blood Pressure [mmHg]	119.19 ± 14.73	-6.56 ± 9.68	115.22 ± 17.07	0.17 ± 10.96	p = 0.058, β = -6.758, SE = 3.288, MD = -6.73 ± 10.38, d = -0.65	p = 0.063, β = -0.235, SE = 0.118	p = 0.457, β = -2.840, SE = 3.715
Diastolic Blood Pressure [mmHg]	78.00 ± 12.52	-3.06 ± 8.96	75.22 ± 11.69	-1.50 ± 7.96	p = 0.715, β = -0.893, SE = 2.406, MD = -1.56 ± 8.44, d = -0.19	**p = 0.002, β = -0.406, SE = 0.115**	p = 0.496, β = -1.892, SE = 2.716
Blood Glucose* [mg/Dl]	101.81 ± 14.30	4.94 ± 21.19	100.89 ± 18.71	3.78 ± 18.48	p = 0.987, β = -0.001, SE = 0.049, MD = 1.16 ± 19.80, d = 0.06	**p = 0.002, β = -0.600, SE = 0.173**	p = 0.634, β = -0.026, SE = 0.053
Waist Circumference [cm]	80.25 ± 10.91	-1.25 ± 2.33	80.03 ± 9.34	0.50 ± 2.09	**p = 0.019, β = -1.861, SE = 0.750, MD = -1.75 ± 2.21, d = -0.79**	p = 0.140, β = -0.059, SE = 0.039	p = 0.602, β = -0.410, SE = 0.777
Bodymass [kg]	79.89 ± 15.40	-0.26 ± 1.15	80.85 ± 14.00	0.19 ± 1.13	p = 0.275, β = -0.460, SE = 0.413, MD = -0.45 ± 1.14, d = -0.40	p = 0.437, β = -0.012, SE = 0.015	p = 0.895, β = 0.057, SE = 0.428
Muscle Mass* [kg]	32.92 ± 7.21	0.51 ± 0.61	33.79 ± 7.00	0.12 ± 1.10	p = 0.146, β = 0.012, SE = 0.008, MD = 0.38 ± 0.90, d = 0.42	p = 0.980, β = -0.000, SE = 0.020	p = 0.953, β = -0.000, SE = 0.008
Fat Mass [%]	17.17 ± 5.82	-1.38 ± 1.71	16.04 ± 5.64	-0.03 ± 2.03	p = 0.055, β = -1.327, SE = 0.664, MD = -1.35 ± 1.89, d = -0.72	p = 0.662, β = 0.027, SE = 0.061	p = 0.391, β = 0.604, SE = 0.692

SD, Standard Deviation; MD, Mean Difference; β, Regression Coefficient (Estimate); SE, Standard Error; d, Cohen’s d effect size; IMTP, Isometric Mid-Thigh Pull; CMJ, Countermovement Jump; WOD, Workout of the Day; BP, Blood Pressure. Indicates variables where log-transformed data were used for the LMM analysis due to violation of normality assumptions. Statistically significant values (p) are highlighted in bold.

Data are presented as baseline values (mean ± standard deviation [SD]) and mean difference (MD ± SD) for each intervention type. The results of the Linear Mixed Model (LMM) analysis are reported for the intervention effect (BP vs. TP), baseline effect, and sequence effect.

### 3.2 Health related outcomes

**Figure 5:**
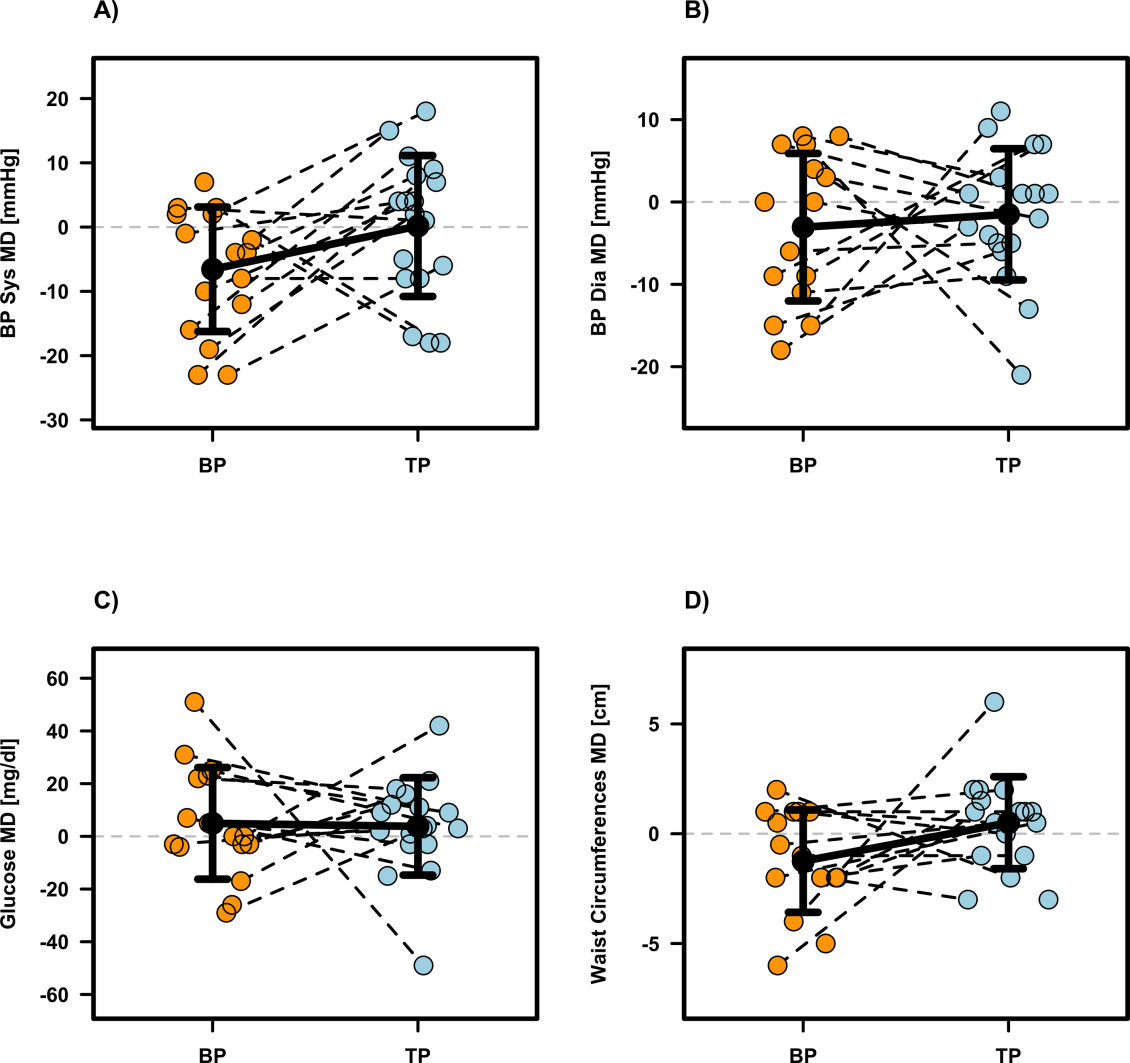
Individual and mean difference (MD) for systolic blood pressure [**(A)**, BP Sys], diastolic blood pressure [**(B)**, BP Dia], blood glucose **(C)** and waist circumferences **(D)**. The plots display individual data points (colored dots) for Block Periodization (BP, orange) and Traditional Periodization (TP, light blue). Black crossbars indicate the group mean difference ± standard deviation.

**Figure 6:**
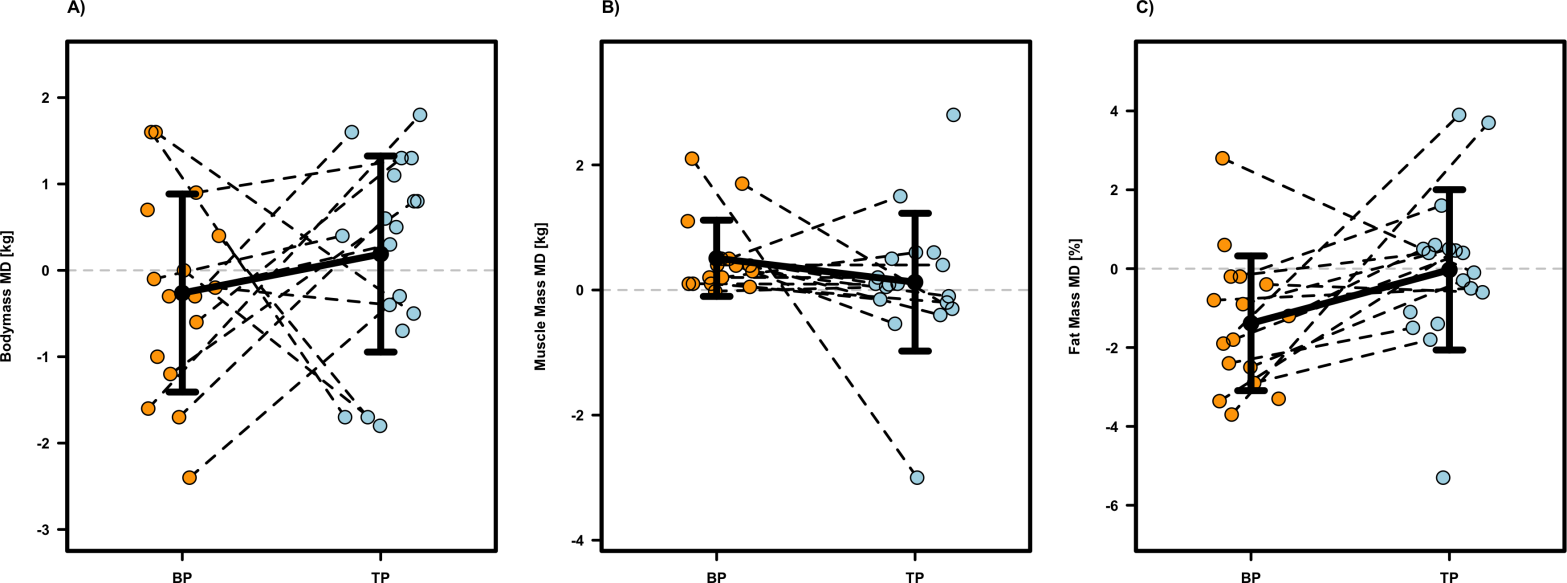
Individual and mean difference (MD) for bodymass **(A)**, muscle mass **(B)** and fat mass **(C)**. The plots display individual data points (colored dots) for Block Periodization (BP, orange) and Traditional Periodization (TP, light blue). Black crossbars indicate the group mean difference ± standard deviation.

Most health related outcomes revealed no significant intervention effects (p ≥0.055, see **Table 1**), except for waist circumference (p = 0.019, β = -1.861, SE = 0.750, MD = -1.75 ± 2.21, d = -0.79) in favor of BP compared to TP. Individual participant data and mean differences are presented in **Figures 5**, **6**. No significant intervention effects but moderate effect sizes in favor auf TP for Systolic Blood Pressure (p = 0.058, β = -6.758, SE = 3.288, MD = -6.73 ± 10.38, d = -0.65) and Fat Mass (p = 0.055, β = -1.327, SE = 0.664, MD = -1.35 ± 1.89, d = -0.72), indicating a trend toward improved metabolic health and body composition. Regarding baseline characteristics, significant effects were detected for Diastolic Blood Pressure (p =0.002, β= −0.406, SE = 0.115) and Blood Glucose (p =0.002, β= −0.600, SE = 0.173), suggesting that participants with higher initial values experienced greater reductions. Apart from these findings, no significant effects for baseline values or the sequence of interventions were found for any other health-related outcome (see **Table 1**).

### 3.3 Monitoring data

**Figure 7:**
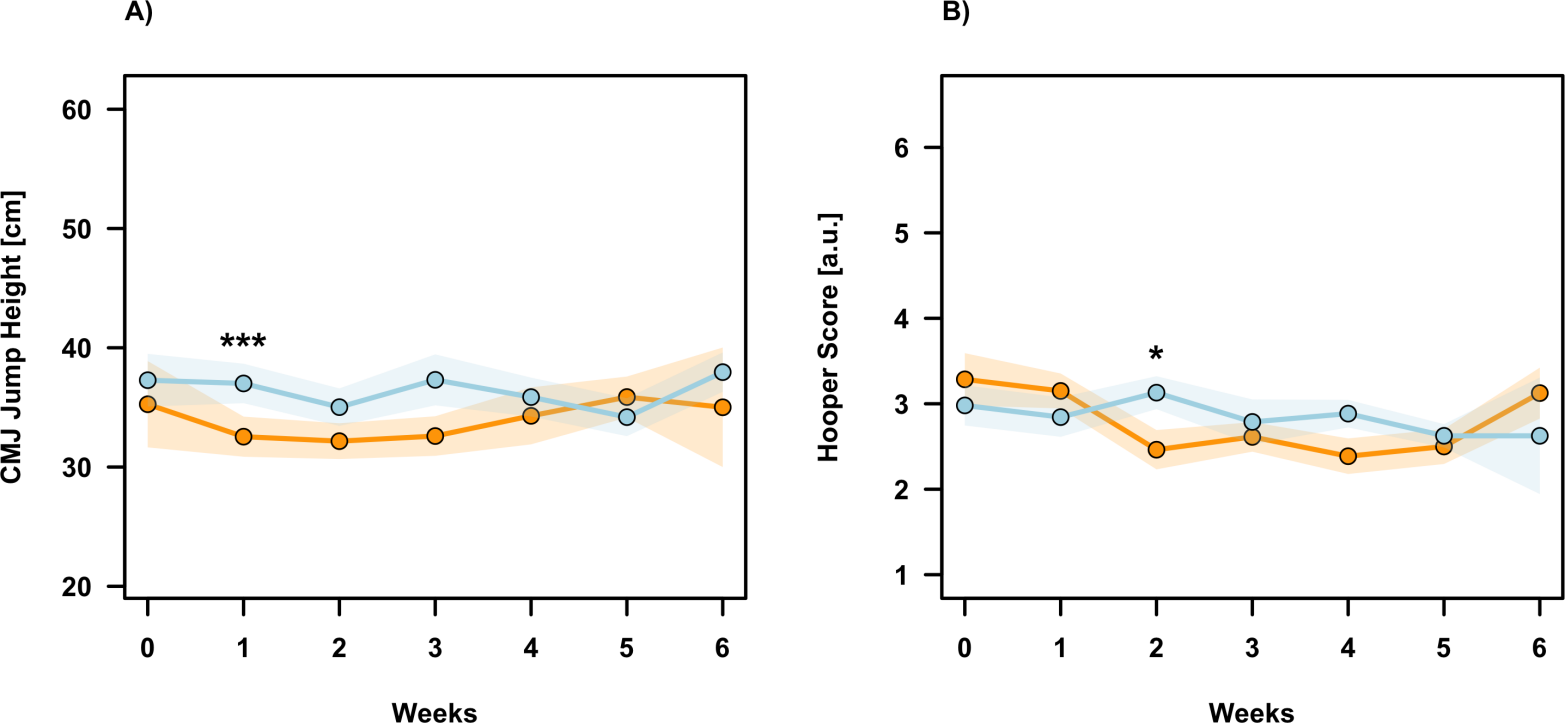
Weekly monitoring of neuromuscular performance and subjective well-being. The panels display the time-course changes for **(A)** Countermovement Jump (CMJ) height and **(B)** the Hooper Index score across the six-week intervention. The Block Periodization group (BP) is depicted in orange, and the Traditional Periodization group (TP) is depicted in light blue. Data points represent the group means, while the shaded regions indicate the standard error (SE). Asterisks denote significant differences between groups derived from *post hoc* analysis (*p < 0.05; ***p < 0.001). a.u., arbitrary units.

For CMJ height, the LMM revealed a significant main effect for Intervention (F = 11.20, p =0.001, η_p_^2^ = 0.06), indicating generally lower jump performance in the BP compared to TP across the monitoring period (**Table 2**). No significant main effect for Time (F = 0.45, p =0.85, η_p_^2^ <0.01) and Intervention × Time interaction (F = 1.90, p =0.083, η_p_^2^ = 0.06) were observed. *Post hoc* analysis revealed significantly lower values for BP compared to TP in Week 1 (p <0.001, MD =−2.84 ± 0.66, d =−1.41). In addition, non-significant differences with moderate effect sizes indicated lower BP performance compared to TP in Week 0 (p =0.106, MD= −1.60 ± 0.98, d =−0.79) and Week 3 (p =0.056, MD =−1.49 ± 0.77, d =−0.74). All other pairwise comparisons remained non-significant (p ≥0.248) (**Figure 7A**).

**Table 2:** Weekly monitoring data of countermovement jump (CMJ) and subjective well-being (Hooper Index) comparing Block Periodization (BP) and Traditional Periodization (TP) groups.

	CMJ [cm]			Hooper [au]		
Week	BP	TP	PostHoc	BP	TP	PostHoc
0	35.27 ± 9.57	37.28 ± 7.64	p =0.106, MD = -1.60 ± 0.98, d =-0.79	3.29 ± 1.15	2.98 ± 0.84	p =0.346, MD = 0.30 ± 0.32, d =0.37
1	32.55 ± 7.33	37.01 ± 7.38	**p < 0.001, MD = -2.84 ± 0.66, d =-1.41**	3.15 ± 1.02	2.84 ± 1.13	p =0.166, MD = 0.33 ± 0.24, d =0.4
2	32.17 ± 5.80	35.02 ± 7.67	p =0.460, MD = -0.52 ± 0.70, d =-0.26	2.46 ± 1.03	3.13 ± 0.96	**p =0.016, MD = -0.61 ± 0.25, d =-0.75**
3	32.60 ± 6.20	37.31 ± 8.26	p =0.056, MD = -1.49 ± 0.77, d =-0.74	2.61 ± 0.77	2.79 ± 1.18	p =0.478, MD = -0.19 ± 0.26, d =-0.23
4	34.29 ± 8.61	35.86 ± 7.64	p =0.798, MD = -0.19 ± 0.74, d =-0.09	2.39 ± 0.98	2.88 ± 0.82	p =0.132, MD = -0.36 ± 0.24, d =-0.45
5	35.87 ± 7.28	34.18 ± 6.75	p =0.639, MD = -0.33 ± 0.69, d =-0.16	2.50 ± 0.98	2.62 ± 0.64	p =0.613, MD = -0.12 ± 0.25, d =-0.15
6	35.01 ± 7.09	37.96 ± 2.85	p =0.248, MD = -2.21 ± 1.91, d =-1.1	3.12 ± 0.60	2.62 ± 1.36	p =0.759, MD = 0.18 ± 0.59, d =0.22

Data are presented as mean ± standard deviation for group descriptive values. Pairwise comparisons are reported as mean difference ± SD derived from linear mixed model *post hoc* analysis. In addition, Cohen’s d as pairwise effect size and *post hoc* significance p-values are given. Statistically significant values (p) are highlighted in bold.

For the Hooper Index, no significant main effect for Intervention was found (F = 0.28, p =0.60, η_p_^2^ <0.01). However, there was a significant main effect for Time (F = 2.65, p =0.017, η_p_^2^ = 0.06), indicating that subjective well-being fluctuated significantly over the course of the study regardless of group allocation. The Intervention × Time interaction was not significant (F = 1.78, p =0.10, η_p_^2^ = 0.04). *Post hoc* analysis revealed significantly lower scores for BP compared to TP in Week 2 (p =0.016, MD =−0.61 ± 0.25, d =−0.75). All other pairwise comparisons were non-significant (p ≥0.132, d ≤0.45) (**Figure 7B**).

On average, the BP group completed 88.1 ± 5.6% of the training sessions, while the TP group completed 86.7 ± 5.6%.

## 4 Discussion

The present study compared the effects of Block Periodization (BP) versus Traditional Periodization (TP) on physical performance and health-related parameters in experienced HIFT athletes using a randomized controlled crossover design. The main finding of our investigation was that BP was associated with more favorable changes in selected maximal strength outcomes, including IMTP, deadlift 1RM, and CrossFit^®^ Total. In contrast, no significant between-condition differences were observed for metabolic conditioning performance. Although we expected the concentrated organization of high-intensity stimuli to support mixed-modal conditioning performance, this was not reflected in significant between-condition differences in metabolic conditioning outcomes. Furthermore, BP was associated with a reduction in waist circumference, despite conflicting data from bioelectrical impedance analysis regarding fat mass. Thus, the findings provide partial support for the assumption that concentrating high-intensity conditioning loads may induce favorable adaptations compared with a more evenly distributed training-intensity structure, but this effect appears to be limited primarily to strength-related outcomes.

These findings suggest that concentrating high-intensity training loads into specific blocks may provide a favorable framework for strength-related adaptations in HIFT athletes, even when total training volume and load are matched between intervention conditions. This outcome aligns with the core principles of Block Periodization, which emphasize the successive, concentrated development of targeted abilities to avoid potential interference effects associated with concurrent training (Issurin, 2008, 2010). Since HIFT, such as CrossFit^®^, inherently combines endurance, gymnastics, and high-load resistance training (Claudino et al., 2018; Dominski et al., 2022; Schlegel, 2020), the risk of such interference (Coffey and Hawley, 2017; Hickson, 1980) is high (Schlegel, 2020).

Furthermore, the significant improvements observed in IMTP, Deadlift 1RM and the CrossFit^®^ Total (the sum of 1RM squat, deadlift, and overhead press) following the BP intervention highlight the effectiveness of this approach for maximizing specific strength capacities. These findings corroborate previous research by Painter et al. (2012), who demonstrated that block periodization models are more effective than daily undulating or traditional models for maximal strength development in trained individuals. Previous literature consistently identifies maximal strength, often measured by 1RM exercises like the squat and deadlift, as crucial physiological predictors of high-level HIFT performance and ranking in CrossFit^®^ open competitions (Butcher et al., 2015; Dexheimer et al., 2019; Mangine et al., 2020; Meier et al., 2023; Serafini et al., 2018; Zeitz et al., 2020). For instance, CrossFit^®^ total is strongly associated with success in Olympic lifts and overall CrossFit^®^ benchmark performance (Meier et al., 2021; Schlegel, 2020). Therefore, the enhanced strength gains achieved through BP are highly relevant to competitive success in this sport. Maximizing maximal strength development, as achieved in the BP phase, provides a foundation for subsequent power expression (Suchomel et al., 2018; Wetmore et al., 2020). While studies in endurance athletes frequently focus on the aerobic benefits of BP (Mølmen et al., 2019), our findings extend the application of BP to multimodal sports, confirming its capacity to promote neuromuscular adaptations that enhance strength related outcomes. In contrast, some studies on periodization in HIFT settings, specifically comparing polarized training to traditional approaches, found similar maximal strength gains between groups over six weeks (Held et al., 2024), suggesting that methodological distinctions in how the ‘block’ is structured critically influence the outcome.

The results regarding metabolic conditioning and the interference effect must be interpreted within the strict constraints of the study design. It is crucial to emphasize that over the full six-weeks intervention both BP and TP groups adhered to an identical total training volume and an identical aggregate Training Intensity Distribution. The differentiating factor was exclusively the temporal organization of these loads. While TP maintained a uniform distribution of high-intensity stimuli throughout the intervention the BP group polarized this distribution by concentrating the highest metabolic loads specifically into weeks one and four while significantly reducing intensity in the remaining weeks. This temporal manipulation may represent one possible strategy to reduce interference-related effects, as endurance-type stimuli have been suggested to activate AMPK-related pathways that may attenuate mTOR signaling involved in strength adaptations (Coffey and Hawley, 2017; Hickson, 1980). In the TP group the constant presence of high-intensity metabolic conditioning likely created a chronic and low-level interference signal whereas the BP approach effectively compartmentalized this stress. By confining the peak metabolic demands to specific shock microcycles the block approach likely created subsequent windows of opportunity in the recovery weeks where neuromuscular adaptations could occur with reduced interference (Issurin, 2008). Although modern HIFT utilizes high-intensity interval modalities that biomechanically resemble resistance training, which may mitigate the classic interference effects observed in running-based protocols (Sabag et al., 2018; Schlegel, 2020), our data suggests that temporal segregation may be advantageous for strength development. Notably, CrossFit^®^-specific performance improved only marginally in both groups despite two consecutive six-week training phases, indicating that the overall training load may have induced a state of functional overreaching rather than pronounced performance enhancement. In this context, block periodization appears not primarily to amplify performance adaptations, but rather to better manage accumulated fatigue, thereby preserving strength expression and facilitating transfer to work capacity. While this strategy did not yield significant group differences in pure endurance tasks like the 1000m rowing time trial, the moderate effect sizes favoring block periodization in the Mixed Modal WOD suggest a valuable transfer of strength to work capacity. Nevertheless, the maintenance of metabolic conditioning following the BP mesocycle, confirms that the strategic concentration of strength work did not compromise the athletes’ endurance capacity. This is critical in HIFT, where benchmark workouts impose extreme metabolic loads, often characterized by maximal heart rates and high blood lactate concentrations (Forte et al., 2022; Jacob et al., 2020). This outcome generally aligns with research in endurance sports. Meta-analyses and intervention studies, predominantly conducted in cyclists and cross-country skiers, often reveal that BP is an adequate alternative training strategy to TP, frequently yielding similar or even slightly superior training effects on maximal oxygen uptake (V̇O2max) and maximal aerobic power (Almquist et al., 2022; Mølmen et al., 2019; Rønnestad et al., 2014). Although some studies applying BP models to endurance training, where high-intensity interval training (HIT) loads were concentrated, showed no additional benefits over evenly distributed HIT models when matching loads (McGawley et al., 2017), the overall trend is that BP does not detract from aerobic gains (Mølmen et al., 2019; Solli et al., 2019). The current study reinforces this notion in the HIFT context: by controlling total training volume of the MetCon and running sessions across the two periodization models, BP may have provided the necessary recovery window during low-intensity weeks to potentiate strength gains without incurring the negative residual effects often associated with excessive chronic concurrent training (Schlegel, 2020).

The finding that BP was associated with a significant reduction in waist circumference may indicate a favorable anthropometric change. Reductions in waist circumference often indicate a decrease in central (visceral) adiposity, a key health parameter (Ross et al., 2020). While the bioelectrical impedance analysis (BIA) data concerning fat mass were inconclusive, possibly due to known limitations in BIA accuracy compared to methods like DEXA or the specific anthropometric protocols used (Matias et al., 2021), the clear reduction in waist circumference suggests positive morphological adaptation. General HIFT participation is known to induce improvements in body composition, including reductions in body fat percentage and increases in lean body mass (Cavedon et al., 2020; Serafini et al., 2018). Furthermore, lower body fat percentage is correlated with higher physical and physiological performance in competitive athletes (Carreker and Grosicki, 2020; Crawford et al., 2011). The structured, concentrated load used in BP may have optimized the anabolic window for muscle and strength gains (McGawley et al., 2017), potentially contributing to a favorable shift in body composition, even if overall fat mass change was difficult to capture precisely with the secondary measurement method.

The monitoring data collected via the CMJ and the Hooper Index (Hooper et al., 1995) provides critical insight into the physiological cost of the BP model. The significant reduction in CMJ height and subjective well-being observed during and immediately after the concentrated loading week in the BP group indicates a state of functional overreaching. This acute fatigue response is a hallmark of successful block periodization, distinguishing it from non-functional overreaching or overtraining. The subsequent return to, and improvement above, baseline levels by the post-testing phase confirms that the prescribed recovery periods were sufficient to allow for proper adaptations (Issurin, 2008, 2010). In contrast, the TP group exhibited almost stable CMJ and Hooper values throughout the intervention, suggesting that the distributed load did not induce a significant alarm phase in these advanced athletes. These findings may validate the utility of the CMJ and Hooper Index as sensitive monitoring tools for detecting accumulated fatigue in HIFT populations, consistent with findings in other team and individual sports (Claudino et al., 2017; Saw et al., 2016).

### 4.1 Limitation

Despite the robust design, this study is subject to specific limitations that warrant discussion. First, the crossover design introduces potential challenges related to sequence and carry-over effects. A significant sequence effect was observed in the Step Test, where the group starting with TP showed greater improvements, suggesting that learning effects may have contributed to changes in this specific test. In addition, previous individual exposure to structured periodization models was not systematically assessed. Therefore, participants may have differed in their familiarity with concentrated or evenly distributed training organization, which may have influenced responsiveness to the interventions. This is particularly relevant in a crossover design, as participants entered the second intervention phase with a different training and adaptation history than at baseline. Future research should incorporate more extensive familiarization sessions to isolate physiological adaptations from coordinative improvements (Currell and Jeukendrup, 2008; Hopkins, 2000). Second, nutritional intake was not strictly controlled during the intervention periods. Given the heavy reliance on glycolytic energy systems during the concentrated blocks, carbohydrate availability plays a crucial role in recovery and performance (Kerksick et al., 2018; Thomas et al., 2016). It is possible that ad libitum eating was insufficient to meet the demands of the BP “shock week”, potentially dampening the magnitude of the positive adaptations or exacerbating fatigue (Escobar et al., 2016; Gogojewicz et al., 2020; Martinho et al., 2025). Future studies should explicitly control or manipulate macronutrient intake to investigate the interaction between nutrition and periodization models. Third, strength training performed in addition to the prescribed intervention was not formally monitored. Although all participants received an identical strength training plan prior to study onset and were instructed to maintain this program throughout the intervention period, adherence, strength training volume, and intensity could not be verified. Consequently, it cannot be determined whether individual participants deviated from the prescribed strength training recommendations by performing greater or lesser volumes than intended. While it is assumed that such deviations were comparable across participants and conditions, this assumption cannot be empirically confirmed. Previous research also indicates that supervised resistance training is associated with higher adherence and superior strength adaptations compared with self-guided approaches (Gavanda et al., 2025), suggesting that variability in supervision exposure may have further contributed to interindividual differences in strength-related outcomes. Fourth, menstrual cycle phase and hormonal contraceptive use were not systematically recorded in female participants. As hormonal fluctuations may influence neuromuscular performance, substrate metabolism, recovery and perceived fatigue, future studies should control or at least document menstrual cycle phase and hormonal contraceptive use when examining training adaptations in mixed-sex HIFT cohorts. Finally, the six-week intervention period, while sufficient for neural strength adaptations, may have been too short to elicit significant structural aerobic changes (e.g., mitochondrial density) in already (highly) trained athletes (Folland and Williams, 2007; Granata et al., 2018).

### 4.2 Conclusion

In conclusion, under the conditions of this randomized crossover study, block periodization was associated with more favorable changes in selected maximal strength outcomes compared with a more evenly distributed traditional periodization model, while metabolic conditioning outcomes were largely maintained. However, given short intervention duration, potential sequence effects and incomplete control of external strength training, these findings should be interpreted cautiously. From a practical perspective, concentrated loading blocks may be considered as a feasible strategy to organize high-intensity training stimuli in experienced HIFT athletes, particularly when strength-related adaptations are targeted. Further longitudinal research using more homogeneous cohorts, longer intervention periods and stricter control of concurrent strength training should explore the optimal sequence and duration of BP blocks to integrate strength and conditioning while systematically tracking performance biomarkers and recovery metrics.

## Data Availability

The raw data supporting the conclusions of this article will be made available by the authors, without undue reservation.
